# Weight Loss Trajectories and Short-Term Prediction in an Online Weight Management Program

**DOI:** 10.3390/nu16081224

**Published:** 2024-04-19

**Authors:** Bingjie Zhou, Susan B. Roberts, Sai Krupa Das, Elena N. Naumova

**Affiliations:** 1 Friedman School of Nutrition Science and Policy, Tufts University, Boston, MA 02111, USA; 2 Geisel School of Medicine, Dartmouth College, Hanover, NH 03755, USA; susan.b.roberts@dartmouth.edu; 3 Jean Mayer USDA Human Nutrition Research Center on Aging, Tufts University, Boston, MA 02111, USA; sai.das@tufts.edu

**Keywords:** weight loss, weight trajectory, longitudinal data, early prediction, sequential modeling

## Abstract

The extent to which early weight loss in behavioral weight control interventions predicts long-term success remains unclear. In this study, we developed an algorithm aimed at classifying weight change trajectories and examined its ability to predict long-term weight loss based on weight early change. We utilized data from 667 de-identified individuals who participated in a commercial weight loss program (Instinct Health Science), comprising 69,363 weight records. Sequential polynomial regression models were employed to classify participants into distinct weight trajectory patterns based on key model parameters. Next, we applied multinomial logistic models to evaluate if early weight loss in the first 14 days and prolonged duration of participation were significantly associated with long-term weight loss patterns. The mean percentage of weight loss was 7.9 ± 5.1% over 133 ± 69 days. Our analysis revealed four main weight loss trajectory patterns: a steady decrease over time (30.6%), a decrease to a plateau with subsequent decline (15.8%), a decrease to a plateau with subsequent increase (46.9%), and no substantial decrease (6.7%). Early weight change rate and total participating duration emerged as significant factors in differentiating long-term weight loss patterns. These findings contribute to support the provision of tailored advice in the early phase of behavioral interventions for weight loss.

## 1. Introduction

The prevalence of obesity has increased by over 50% globally in the past 35 years and now affects approximately 13% of the world’s population [1]. The United States has one of the highest rates of obesity, over 40% [2] of the adult population. Behavioral lifestyle interventions are recommended as the first-line treatment for individuals with obesity and, in adherent participants, can support >5% weight loss [3,4,5,6]. A range of 5–10% weight loss has been found to be associated with significantly improving metabolic function [7], blood pressure, and HDL cholesterol [8], as well as the potential reversal of type 2 diabetes [9]. However, many participants have low adherence and mean weight loss in nationally scaled programs has been only 3.5% [10]. These findings highlight the need to identify participants who have low adherence early on to add program elements to support further success.

A growing body of research has employed trajectory modeling approaches, such as latent class and growth mixture modeling [11,12,13,14], to classify participants with overweight or obesity into distinct groups based on their weight loss trajectory patterns. This approach can potentially be used to predict longitudinal weight loss patterns among individuals while accounting for individual variability in response to different interventions. In addition, weight loss patterns identified in studies have been found to be associated with characteristics like age, starting BMI [14], and diet adherence [15,16] with weight change patterns. However, commonly used trajectory analyses in previous body weight studies are based on data from research studies and are not compatible with the typical real-world experience of weight monitoring in commercial and community intervention programs, where individuals have varying durations, irregularities, and frequencies of weight recording.

While investigations have shed light on the correlation between initial weight loss and long-term outcomes, the precise duration of the “early” phase or the “early weight loss response” remains unclear [17]. Most studies have found a link between weight loss during the first month of an intervention and weight loss over 3–12 months [18,19,20,21]. However, investigations of the longer-term impact of weight loss within the earliest one or two weeks remain relatively scarce in the literature. To our knowledge, only one study found that very early weight loss within two weeks can predict weight loss up to 6 months but failed to predict weight loss beyond one year [22]. Importantly, this specific study exclusively focused on veterans, and its approach relied on 0.5% and 5% thresholds to define early responders and successful weight loss. However, this method lacked the incorporation of individual heterogeneity and the intricate, non-linear changes in weight loss over extended periods. While the significance of timely intervention adjustment has been recognized, the optimal time threshold to predict subsequent weight loss patterns has yet to be determined.

The primary aim of this study was to develop an algorithm to identify common patterns of weight change and classify participants’ weight trajectories with a method enabling accommodation of variable intervention duration and variable frequency of self-reporting, using 69,363 weight records over one year from 667 participants enrolled in a commercial weight loss program. We also used the approach we developed to examine whether weight records in the first 14 days can predict long-term weight loss patterns.

## 2. Materials and Methods

### 2.1. Study Population

A commercial weight loss program offering clinically impactful behavioral support provided weight data for unrestricted use to Tufts University (Instinct Health Science, www.theidiet.com, accessed on 11 April 2024). The iDiet was developed by co-author SBR, and detailed descriptions of this program are given elsewhere [23,24]. In brief, the iDiet program targets a weight loss of 0.5–1 kg of weight per week by reducing energy intake by 500–1000 calories per day. In addition, it has the specific goal of reducing hunger and food cravings by providing menus and recipes relatively high in dietary fiber and protein and low in energy density and carbohydrate glycemic index. Each program block was 11 weeks long, but individuals could choose to continue with one or more additional blocks. Each 11-week program was implemented as a small group program that met once a week for an hour to learn about nutrition and weight management, discuss it, and support one another. Participants were encouraged but not required to enter their weight records via the program website. The analysis of the iDiet data was approved by the Institutional Review Board at Tufts University.

**Figure 1:**
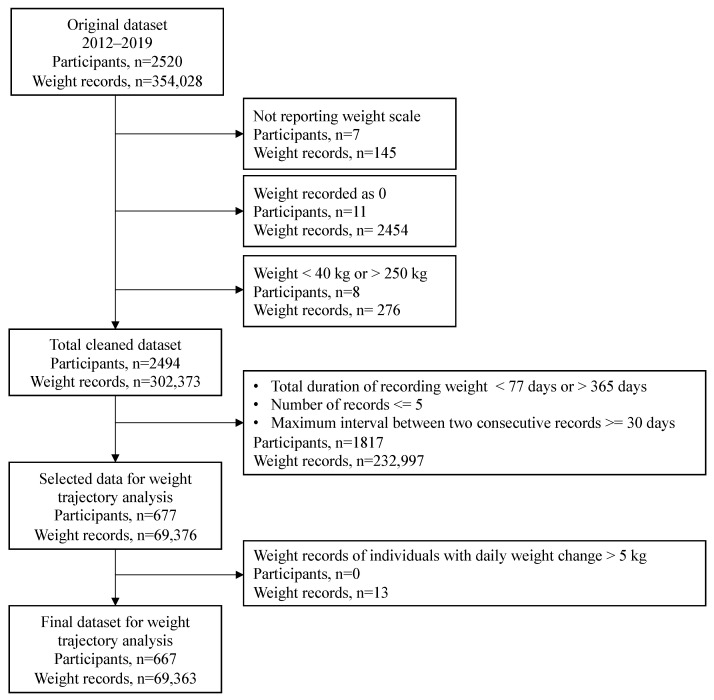
Consort diagram illustrating the selection of study participants.

The original dataset comprised 305,248 weight records from 2520 participants who were enrolled in the program between 2012 and 2019. To ensure data quality, we conducted initial data screening by excluding records based on the following criteria: (1) weight records without reporting the type of unit (kg or lb.), (2) weight values recorded as 0, and (3) weight values lower than 40 kg or higher than 250 kg. Subsequently, the screened dataset contained 302,373 weight records from 2494 participants. For algorithm development and subsequent analysis, we focused on participants who recorded their weight for at least 11 weeks (77 days) and limited the duration between the first and last records to at most one year (365 days), even if they recorded weight for over one year. Additionally, we excluded participants if two consecutive weight records were more than 30 days apart and if they recorded fewer than five weights throughout their program participation. These selection criteria resulted in a study sample of 667 participants, with a total of 69,363 weight records. The process of data selection is depicted in the consort diagram (Figure 1), and comprehensive summary information for both the original and the study sample is given in Table 1. We did not include gender, age, and height information in our data analysis, as these variables had more than 40% missingness in the original datasets, which resulted in less than 50% of participants with complete demographical data. Detailed summary statistics of age and sex information are listed in Table 2.

### 2.2. Sequential Modeling

We employed a sequential polynomial regression modeling approach to analyze individual weight trajectories. This involved fitting three models for each trajectory, starting with a linear term, followed by the addition of a quadratic term, and finally incorporating a cubic term. The model equations for the linear (m_1_), quadratic (m_2_), and cubic (m_3_) models are presented below:

(1) $$m_{1}:\;{\;y}_{i,m_{1},t}=\beta_{0,{i,m}_{1}}+\beta_{1,{i,m}_{1}}*t+\varepsilon_{i,m_{1}}$$

(2) $$m_{2}:\;y_{i,m_{2,t}}=\beta_{0,{i,m}_{2}}+\beta_{1,{i,m}_{2}}*t+\beta_{2,{i,m}_{2}}*t^{2}+\varepsilon_{i,m_{2}}$$

(3) $$m_{3}:\;y_{i,m_{3},t}=\beta_{0,i,m_{3}}+\beta_{1,{i,m}_{3}}*t+\beta_{2,{i,m}_{3}}*t^{2}+\beta_{3,i,m_{3}}*t^{3}+\varepsilon_{i,m_{3}}$$

where $y_{i,t}$ represents the weight value for *i*-person, recorded on *t-*day from the start of individual recording, with *t* ranging from 1 to the last day ($t_{z}$) of recording of each participant; *β*_0_*_,i_* represents the intercept for the *i*-person, *β*_1_*_,i_* represents the coefficient of the linear term *t* for the *i*-person, *β*_2_*_,i_* represents the coefficient of the quadratic term *t*^2^ for the *i*-th person, and β_3,*i*_ represents the coefficient of the cubic term *t*^3^ for the *i*-person; ε_i_ is the error term in the model for the *i*-person.

### 2.3. Weight Trajectory Classification Algorithm

The first step in classifying weight trajectory is to determine the optimal model for each participant. We extracted the adjusted R^2^ of the linear, quadratic, and cubic models to compare the models’ performance for each individual trajectory. A threshold of a 5% change in adjusted R^2^ was applied to assess the superiority of a more complex model over a simpler one. If the percentage change in adjusted R^2^ when comparing the more complex model to the simpler model exceeded 5%, we considered the trajectory to be better fitted by the more complex model. Conversely, if the change did not surpass the 5% threshold, the trajectory was classified using the simpler model.

Subsequently, we estimated a set of key parameters to facilitate further classification: Total duration is denoted as $t_{total,i}=t_{1,i}-t_{z,i}$, where t*_1,i_* and t*_z,i_* represent the first day and the last day of weight recording for the *i*-person.

Predicted weight values on the first day are denoted as ${y*}_{m_{1,}t_{1,i}}$, ${y*}_{m_{2,}t_{1,i}}$, ${y*}_{m_{3,}t_{1,i}}$, and on the last day are denoted as ${y*}_{m_{1,}t_{z,i}}$, ${y*}_{m_{2,}t_{z,i}}$, ${y*}_{m_{3,}t_{z,i}}$, for model m_1_, m_2_ and m_3_, respectively.

For a quadratic model m_2_, the nadir point of the trajectory curve is calculated as:

$${nadir}_{i}=\frac{-2\beta_{2,{i,m}_{2}}}{\beta_{1,{i,m}_{2}}}$$

The predicted weight at the nadir is denoted as ${y*}_{m_{2,}t_{nadir,i}}$.

For a cubic model m_3_, the Δ of the derivative of the cubic model is calculated as:

$${D\;}_{i}=4*{\beta_{2,{i,m}_{3}}}^{2}-12{(\beta }_{1,{i,m}_{3}}*\beta_{3,{i,m}_{3}}).$$

When Δ > 0, there are two vertex points,

$$x_{1,i}=\frac{-\beta_{2,{i,m}_{3}}-\sqrt{{\beta_{2,{i,m}_{3}}}^{2}-4*\beta_{3,i,m_{3}}*\beta_{1,{i,m}_{3}}}}{3*\beta_{3,i,m_{3}}},x_{2,i}=\frac{-\beta_{2,{i,m}_{3}}+\sqrt{{\beta_{2,{i,m}_{3}}}^{2}-4*\beta_{3,i,m_{3}}*\beta_{1,{i,m}_{3}}}}{3*\beta_{3,i,m_{3}}},$$

and the predicted weight of the two vertex points are ${y*}_{m_{3,}x_{1,i}}$ and ${y*}_{m_{3,}x_{2,i}}$, respectively.

After the participant data were grouped into the optimal models, we developed different criteria for the linear, quadratic, and cubic models, respectively, by applying those key parameters above together with the model coefficients to further group participants’ trajectories according to the shape of the individual trajectories. Detailed descriptions of the criteria are listed in Table 3.

Next, we created a multi-panel scatter plot to visually compare the similarities within the same pattern and the differences among different patterns. The scatter plot depicted the individuals’ weight change compared to their previous weight records over time, from the participant’s first day of records and up to one year. The participants classified into the same pattern were grouped into the same panel, so the different panels represented different weight trajectory patterns. The points on the scatter plot are the individuals’ weight records. We also added a Loess smooth curve to each pattern to represent the overall weight change pattern over time. To enhance the utilization of our sequential modeling and its associated parameters, we developed a growth chart-style plot that incorporates additional percentile lines. These percentile lines, namely the 1st, 5th, 10th, 25th, 50th, 75th, 90th, 95th, and 99th, are dynamically determined based on the values of the model coefficients within each pattern. This visualization effectively portrays the predicted weight trajectory over a 14-week period in seven distinct patterns.

### 2.4. Early Prediction Modeling

To examine the association between weight change rate in the first 14 days and the individuals’ trajectory patterns, we applied a multinomial logistic regression adjustment for the number of records in the first 14 days and the total participated durations.

$$log\left(\frac{p_{j}\left(x\right)}{p_{J}\left(x\right)}\right)=\beta_{0,j}+\beta_{1,j}*X_{1,i}+\beta_{2,j}*X_{2,i}+\beta_{3,j}*X_{3,i}$$

where *j* represents a defined weight trajectory pattern, and *j* = 1, *X*_1,*i*_ represents the weight change in the first 14 days (difference divided by 14 days), X_2,*i*_ is number of records collected in the first 14 days, and X_3,*i*_ is the duration, or the number of participated days for *i*-participant.

To examine if early weight change characteristics differ in different trajectory patterns, we extracted the estimates of beta coefficients and standard error from the model output for each pattern compared to a reference, Pattern 1, and exponentiated model coefficients to calculate the odds ratio and 95% confidence interval. The odds ratio estimates the ratio of the likelihood of being in one pattern to the likelihood of being in a reference pattern for every one-unit increase in weight change rate in the first 14 days, adjusting for the number of records in the 14 days of the total participated duration. To improve the model sensitivity, we combined the patterns with low representation.

### 2.5. Model Validation and Sensitivity Analysis

To validate our sequential model, we employed 10-fold cross-validation across all the linear, quadratic, and cubic models. The data were randomly partitioned into 10 equal-sized folds, with 9 folds used for training the model, and the remaining fold used for testing. This process was repeated 10 times, with each fold as the testing set once. We report the root mean squared error (RMSE), and R squared as our primary performance metric. To evaluate the robustness of our classification methodology, we conducted sensitivity analyses. These analyses involved varying the threshold number of classification criteria to examine the stability of the participants’ weight trajectory groupings. Specifically, we tested threshold values 0.5, 1.5, and 2 for C1.1, C2.1, and C3.1, values 1.5 and 2.5 for C2.4, and 2.5 and 3.5 for C3.7. The percentage of the participants who changed their weight trajectory groups was represented as the sensitivity metric. We further conducted a sensitivity analysis to assess the potential impact of age and sex on the prediction model. We additionally included the variables age and sex in the model and compared the change of coefficient among other predictor variables.

All the statistical analyses were conducted using R version 4.2.0. ANOVA was employed to test for differences among groups. Tukey’s honestly significant difference test was used to determine pairwise differences between groups. Statistical significance was established at *p*  <  0.05.

## 3. Results

### 3.1. Pattern Classification

**Figure 2:**
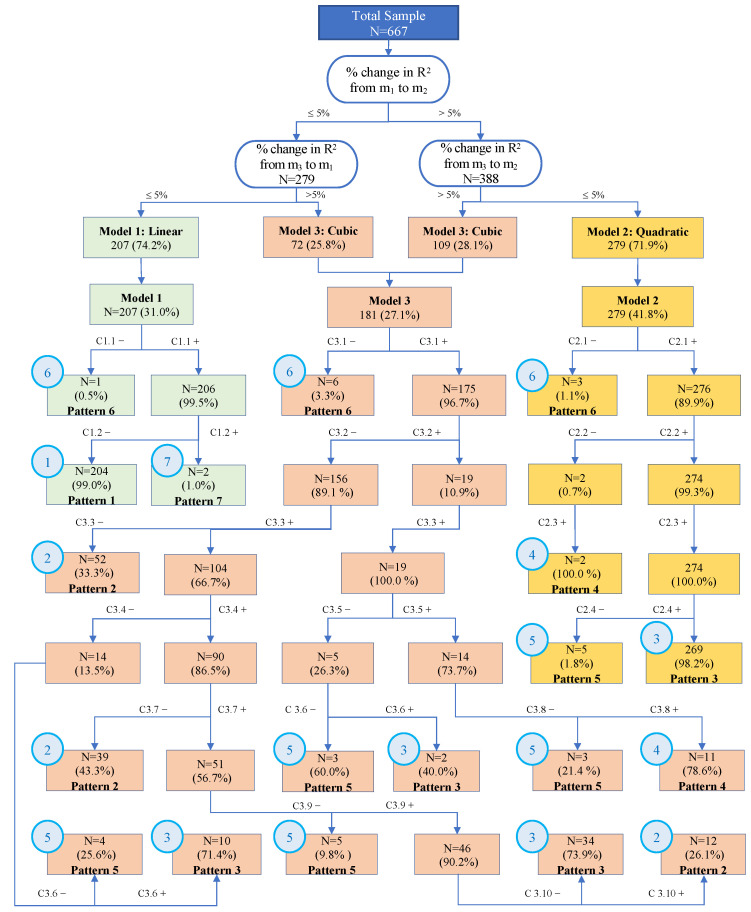
The classification flow diagram demonstrates the sequential classification based on the developed criteria. This diagram illustrates the sequential decision process implemented by our classification algorithm. The first classification step is based on the model’s performance, followed by the customization of criteria for the three different models. The arrows and boxes show how participants are distributed in each step according to the specific criteria employed. Detailed criteria can be found in Table 2. To facilitate clarity, three distinct colors are used to differentiate participants classified into three different models. The light green quadratic represents the classification steps for the linear model, the yellow color corresponds to the quadratic model, and the light orange-pink color represents the cubic model. The number and percentage displayed within each box denote the absolute count and proportion of participants classified into that specific box from the previous upper-layer box. A pattern label is assigned to a box when it reaches a final step that sufficiently identifies a distinct pattern, indicating that the participants within that pattern share a common weight trajectory shape.

We developed a sequential classification process that integrates sequential modeling and classification based on the model parameters. The comprehensive workflow is illustrated in Figure 2. By applying a series of specific criteria, we identified seven distinct weight change patterns, which were sequentially classified. This means that the specific patterns were not determined at the final step of the process but rather through a predetermined sequence of stages and branches during the classification process.

In this process, each individual trajectory underwent rigorous testing against specific criteria in order to assign the pattern with the best fit. For instance, the simple linear fit of Pattern 1, characterized by a steady decline, was detected at the earliest stages of the modeling. The quadratic term, or second-order polynomial, representing a monotonic acceleration or deceleration, required additional verification steps. The cubic, or the third-order polynomial term, was needed to detect patterns with varying trajectories, where more steps were required to complete the classification.

The branching process reflects the likelihood or sufficiency of a select component (linear, quadratic, or cubic) to depict the complexity of an individual trajectory. The branching process within the classification reflects the likelihood or sufficiency of a particular component (linear, quadratic, or cubic) in capturing the complexity of an individual trajectory.

**Figure 3:**
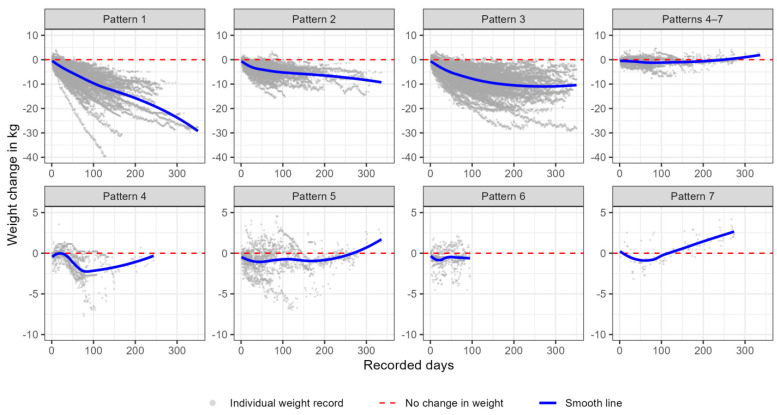
A multi-panel graph describing the weight trajectories among the seven patterns. This is an eight-panel graph in which we grouped participants into seven patterns, according to our sequential classification algorithm, and combined Patterns 4−7 due to low representation. We plotted individual daily weight records in gray dots and a Loess smooth line colored in blue to depict the similar weight change curve within each pattern. The horizontal red dashed line shows zero change over time.

We grouped participants by identifying their trajectories according to the emergent patterns and summarized the weight trajectory characteristics in these patterns. Figure 3 shows the individual trajectories and their representative weight trajectory for each detected pattern. To ease the comparison across the patterns, we plotted the individual weight change over time in multi-panel plots. As our classification criteria are based on the similarity in the shape of the individual’s weight loss trajectories, we observe that each panel shows a distinct change pattern. The density of the records indicates that most participants were grouped into the first three patterns, all of which had a decreased weight over time. The participants in Pattern 1 had the most significant weight loss, and participants in Pattern 3 had the most prolonged participation duration. The last four patterns had much fewer weight records and shorter durations, and people comprising Pattern 6 had the shortest duration of weight recording.

The summary of how participants grouped into different patterns based on the fitted model results is shown in Table 4. The seven patterns were defined as follows: Pattern 1—steady decrease over time (30.6%); 2—decrease to a plateau with the subsequent decline (15.4%); 3—decrease to a plateau with subsequent increase (47.2%); 4—short-term increase at the start followed by a decrease (1.9%); 5—decrease with a prominent increase at the end (3.0%); 6—no detectable increase or decrease (1.5%); and 7—steady increase over time (0.3%). Patterns 1, 2, and 3 accounted for more than 90% of the participants. The trajectories in Pattern 1 and Pattern 7 were classified as such using primarily the results of Model 1, as described by the classification flow diagram (Figure 2).

Patterns 4–7 were consolidated into a single category, referred to as “no substantial decrease” (6.7%) due to low representation. The weight loss characteristics for each pattern are summarized in Table 5. The first three patterns exhibited trajectories with weight loss exceeding 5%. Among these, Pattern 1 displayed the most significant weight loss, accounting for approximately 10% of the initial weight, with an average absolute weight loss of 9.4 kg. In comparison to Pattern 1, Pattern 3 demonstrated a notably longer total duration and a higher number of weight records, with an additional duration of approximately 40 days and approximately 30 more weight records than Pattern 1. Additionally, Pattern 3 exhibited significantly longer gaps between two consecutive weight records when compared to Pattern 1. Pattern 4 experienced <1% mean weight loss, anticipating little or no clinical benefit from reduced body fatness.

### 3.2. Early-Term Prediction

**Figure 4:**
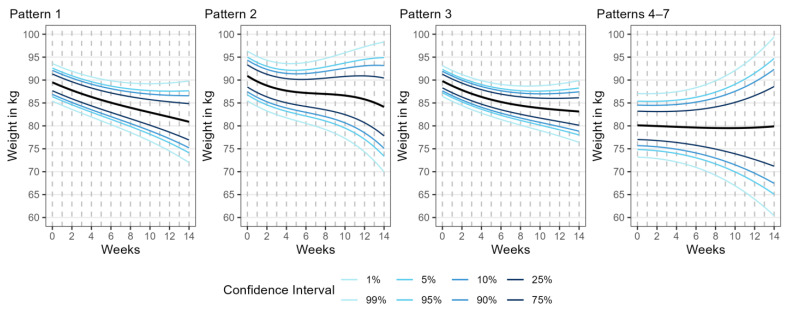
A multiple-panel line graph showing the weight trajectory prediction in four different patterns over the first 14 weeks. The *x*-axis is the weight record time and the time participating in the program in weeks. The *y*-axis is the individual’s daily weight in kilograms. The prediction line is graphed based on the model parameter of the cubic model for each pattern. We further added the 1st, 5th, 10th, 25th, 50th, 75th, 90th, 95th, and 99th percentile confidence interval lines to provide referenced distribution information.

For each pattern, we generated predicted weight loss trajectories for the initial 14-week period to serve as a reference growth chart-style plot. To provide a comprehensive representation of the uncertainty in the predictions, we added confidence interval lines for the 1st, 5th, 10th, 25th, 50th, 75th, 90th, 95th, and 99th percentiles (Figure 4).

We employed a multinomial logistic model to investigate whether early weight change, the number of records, and participating duration could predict the weight loss trajectory patterns over an extended duration, with Pattern 1 serving as the reference category. Our multinomial logistic model analysis yielded several key results. The weight change within the first 14 days is statistically indistinguishable for Patterns 1, 2, and 3. The early change, however, allowed us to differentiate consolidated Patterns 4–7 from Pattern 1. A prolonged duration of participation is a significant distinct feature of Pattern 3 compared to Pattern 1. This suggests that participation in the program for an extended period helps to maintain weight loss. The summary of model results is shown in Table 6.

In validating our sequential model through 10-fold cross-validation, we extracted the mean RMSE and R-squared of the 10-fold cross-validation from the best of the three models of all the participants, with each participant having a mean RMSE and a mean R-squared value. Across all the participants, the average mean RMSE was 0.56, and the average standard deviation of RMSE was 0.16. Moreover, the average mean R-squared was 0.86, and the average standard deviation of R-squared was 0.08. In assessing the model’s sensitivity, the threshold values tested for 0.5 (C1.1, C2.1, and C3.1), 1.5 (C1.1, C2.1, and C3.1), and 2 (C1.1, C2.1, and C3.1) yielded percentage changes in weight trajectory groupings of 2.9%, 6.0%, and 11.4%, respectively. For criterion C2.4, the tested threshold values of 1.5 and 2.5 resulted in changes of 7.3% and 0.3%. For C3.7, the threshold values of 2.5 and 3.5 were associated with changes of 1.0% and 0%. Lastly, the inclusion of age and sex did not significantly change the model results of other variables.

## 4. Discussion

In this study, a novel sequential algorithm was developed that was able to identify four discrete weight trajectory patterns for participants enrolled in a web-based weight management program. An important feature of the algorithm was that it allowed for variable weight frequency and variable program length, thus having utility for commercial and self-determined weight loss initiatives that do not have the regimented data structure of a research trial. Overall, our analysis found that 90% of the participants were categorized into the first three weight loss patterns, including the steady decrease over time, the decrease to a plateau with the subsequent decline, and the decrease to a plateau with subsequent increase. Using the algorithm classification system, weight measures during just the first two weeks of weight loss were able to classify individuals as having successful or unsuccessful weight loss over 12 months, identifying a new potential way to tailor weight loss recommendations to the individual at an earlier point in the intervention or program to maximize success.

Previous studies evaluating weight trajectories in randomized controlled trials have identified between two and seven weight change patterns [11,12,14] or lifestyle interventions in real-world settings [12,14,15,24,25,26]. Three patterns of weight loss are commonly reported: modest, moderate, and substantial weight loss [13,14], with some studies describing additional patterns related to weight regain; if more than three patterns were discovered in studies, they were either weight increases or no change in weight [16,25,27]. Our findings are consistent with previous research in that most of our participants fit into one of the three effective weight loss patterns. In addition, our study delved into a more intricate weight record data structure, which contained a broader range of weight record counts, variable participation durations, and irregular time intervals. Nevertheless, our methodology demonstrated the ability to accommodate substantial and irregular datasets. Notably, this methodology not only classified participants based on the magnitude of weight loss but also factored in their temporal engagement with the program.

Several studies have suggested that significant early weight loss predicts long-term weight loss success 1–2 years after lifestyle intervention programs [14,15,25]. However, these analyses used weight information in the first 1–3 months of weight loss, when most weight loss occurs [20,28,29]. Here, we show that weight loss trajectories in just the first 14 days of a behavioral program can predict weight loss patterns. Thus, similar to previous studies, we found a higher chance of successful weight loss related to a higher weight loss rate in the early period, but our shorter identification period allows for significantly more rapid help to address low participant adherence. Specifically, weight loss at 14 days significantly predicted whether the individual would be categorized in Patterns 1, 2, or 3, all with clinically impactful weight loss, versus Pattern 4 with <1% mean weight loss. This observation is consistent with the findings of one prior study reporting that weight loss within the two-week timeframe predicts weight loss at 6 months [22] and extends that finding by showing that this categorization successfully predicts weight loss success to 12 months. In addition, our approach demonstrated the potential to predict weight status at any temporal juncture. This information can help clinicians and counselors offer timely suggestions to participants and improve the efficacy of weight management programs.

Our study has limitations. First, we developed the algorithm using exclusively weight records. Further analysis could enhance the approach by including other characteristics of participants (such as body mass index, age, medical history, diet, smoking habits, alcohol consumption, etc.) when available [30]. Our classification algorithm could also potentially be more precise if we had weight pattern information before weight loss program initiation, or early history of weight fluctuations [31]. This information will allow us to examine the before- and after-effects using segmented modeling [32], explore seasonal and event-specific variations over time [33], or consider personalized records. In addition, our sample data participants were predominantly middle-aged women from a commercial weight loss program who might have strong motivations to lose weight, reducing the generalizability of results to other populations and settings. Future research is warranted to validate our findings across diverse datasets, including those from non-commercial weight loss interventions and programs with different structures and participant demographics. Further research could also explore the comparison between the clustering results obtained from the proposed algorithm and alternative clustering methods, such as growth mixture models and latent growth models. Specifically, investigating the applicability of different models in flexibly handling irregular data with varying measurement frequencies and assessing their effectiveness in classifying and predicting weight loss would be valuable. By using the fundamental functional forms to weight trajectories that allow for considering inertia-prone processes for irregularly spaced records, we could ensure the broad applicability of the algorithm as a machine learning tool.

## 5. Conclusions

In summary, the weight trajectories and prediction charts provided in this study indicate a way to support lifestyle interventions for weight loss by using weight data during just the first 14 days to predict likely success. Sequential predictive modeling of weight change patterns can be expanded with additional datasets, where body weight information is requested daily to help inform personalized weight management programs.

## Figures and Tables

**Table 1:** Summary statistics for weight change characteristics in whole and study samples.

	Mean	SD	Q1	Median	Q3	Min	Max
Whole Sample ^a^							
Duration (days)	265	377	49	89	333	1	2537
Number of records	121	184	25	64	137	1	2090
Longest interval (days)	86.5	187	5	12	56	0	2030
Initial weight (kg)	89.4	24.4	73	85.3	100	44.9	250
Weight loss (kg)	4.7	6.9	1	3.4	7	−126	99.7
Weight loss (%)	5.1	7	1.3	4	8.1	−134	56.2
Study Sample ^b^							
Duration (days)	133	68.7	83	101	168	77	365
Number of records	104	60.7	67	84	127	15	357
Longest interval (days)	9.8	7.6	3	8	14	1	29
Initial weight (kg)	89.4	22.5	74	85.7	99.7	48.1	243
Weight loss (kg)	7.1	5.3	3.7	6.3	9.5	−3.7	52.3
Weight loss (%)	7.9	5.1	4.4	7.2	10.3	−3.9	32.3

^a^: Whole sample: 2494 participants and 302,373 records. ^b^: Study sample: 667 participants and 69,363 records.

**Table 2:** Summary statistics for age and sex in whole and study samples.

		Whole Sample ^a^	Study Sample ^b^
Age (years)	Mean	52.6	53.3
	SD	12.7	12.5
	Q1	43.0	45.3
	Median	54.0	54.0
	Min	21.0	22.0
	Max	87.0	81.0
	NA’s ^c^	1171 (47.0%)	381 (57.1%)
Sex (n, %)	Female	1242 (49.8%)	259 (38.8%)
	Male	240 (9.6%)	56 (8.4%)
	NA’s ^c^	1012 (40.6%)	352 (52.8%)

^a^: Whole sample: 2494 participants and 302,373 records. ^b^: Study sample: 667 participants and 69,363 records. ^c^: NA’s refer to missing values within the dataset.

**Table 3:** Annotated formulas of classification criteria by models.

Model	Criteria	Criteria−	Criteria+
m_1_	C1.1	${y*}_{m_{1,}t_{z,i}}-{y*}_{m_{1,}t_{1,i}}\leq 1$	${y*}_{m_{1,}t_{z,i}}-{y*}_{m_{1,}t_{1,i}}>1$
m_1_	C1.2	$\beta_{1,m_{1},i}\leq 0$	$\beta_{1,m_{1},i}>0$
m_2_	C2.1	$\left|{y*}_{m_{2,}t_{1,i}}-{y*}_{m_{2,}t_{nadir,i}}\right|\leq 1$ and $\left|{y*}_{m_{2,}t_{z,i}}-{y*}_{m_{2,}t_{nadir,i}}\right|\leq 1$	$\left|{y*}_{m_{2,}t_{1,i}}-{y*}_{m_{2,}t_{nadir,i}}\right|>1$ or $\left|{y*}_{m_{2,}t_{z,i}}-{y*}_{m_{2,}t_{nadir,i}}\right|>1$
m_2_	C2.2	$\beta_{2,{i,m}_{2}}\leq 0$	$\beta_{2,{i,m}_{2}}>0$
m_2_	C2.3	${nadir}_{i}\leq 0$	${nadir}_{i}>0$
m_2_	C2.4	${nadir}_{i}\leq t_{total,i}/2$	${nadir}_{i}>t_{total,i}/2$
m_3_	C3.1	$\left|{y*}_{m_{3,}t_{1,i}}-{y*}_{m_{3,}t_{z,i}}\right|\leq 1$ and $\left|{y*}_{m_{3,}x_{1,i}}-{y*}_{m_{3,}x_{2,i}}\right|\leq 1$	$\left|{y*}_{m_{3,}t_{1,i}}-{y*}_{m_{3,}t_{z,i}}\right|>1$ or $\left|{y*}_{m_{3,}x_{1,i}}-{y*}_{m_{3,}x_{2,i}}\right|>1$
m_3_	C3.2	$\beta_{3,i,m_{3}}\leq 0$	$\beta_{3,i,m_{3}}>0$
m_3_	C3.3	$D_{i}\leq 0$	$D_{i}>0$
m_3_	C3.4	$t_{total,i}\leq x_{1,i}$	$t_{total,i}>x_{1,i}$
m_3_	C3.5	$x_{1,i}\leq 0$	$x_{1,i}>0$
m_3_	C3.6	${y*}_{i,m_{3,}t_{1}}-{y*}_{i,m_{3,}t_{z}}\leq 0$	${y*}_{i,m_{3,}t_{1}}-{y*}_{i,m_{3,}t_{z}}>0$
m_3_	C3.7	$x_{1,i}{-x}_{2,i}{\leq t}_{total,i}/3$	$x_{1,i}{-x}_{2,i}{>t}_{total,i}/3$
m_3_	C3.8	${y*}_{m_{3,}x_{1,i}}-{y*}_{m_{3,}t_{z,i}}\leq 0$	${y*}_{m_{3,}x_{1,i}}-{y*}_{m_{3,}t_{z,i}}>0$
m_3_	C3.9	${y*}_{m_{3,}t_{1,i}}-{y*}_{m_{3,}x_{1,i}}\leq 0$	${y*}_{m_{3,}t_{1,i}}-{y*}_{m_{3,}x_{1,i}}>0$
m_3_	C3.10	${y*}_{m_{3,}x_{1,i}}-{y*}_{m_{3,}t_{z,i}}\leq 0$	${y*}_{m_{3,}x_{1,i}}-{y*}_{m_{3,}t_{z,i}}>0$

**Table 4:** Weight trajectory pattern description and assignment based on sequential polynomial regression.

Pattern	Description	m_1_	m_2_	m_3_	Total	Percentage
1	Steady decrease over time	204	-	-	204	30.6%
2	Decrease to a plateau with subsequent decline	-	-	103	103	15.4%
3	Decrease to a plateau with subsequent increase	-	269	46	315	47.2%
4–7	No substantial decrease	3	10	32	45	6.7%
	Total (Patterns 1–7)	207	279	181	667	100.0%
4	Short-term increase at the start followed by decrease	-	7	14	13	1.9%
5	Decrease with a prominent increase at the end	-	-	12	20	3.0%
6	No detectable increase or decrease	1	3	6	10	1.5%
7	Steady increase over time	2	-	-	2	0.3%

**Table 5:** Summary of weight loss, initial weight, participating duration, and number of weight records by pattern.

Pattern	Mean	SD	Q25	Median	Q75	Min	Max
Weight loss (%)							
Pattern 1	10.3	5.4	6.7	9.2	13	1.5	32.3
Pattern 2	6.4 *	3.1	4.2	5.7	7.9	0.7	18.5
Pattern 3	7.8 *	4.7	4.4	7.1	10.1	−1.6	28.3
Pattern 4	3.0 *	2.1	1.2	2.9	4.5	0.5	6.9
Pattern 5	−0.2 *	1.3	−0.9	−0.1	0.4	−3.9	1.8
Pattern 6	1.0 *	1.1	0.5	1.3	1.5	−1.0	2.9
Pattern 7	−2.9 *	0.5	−3.1	−2.9	−2.8	−3.3	−2.6
Patterns 4–7	0.9 *	2.2	−0.4	0.5	1.8	−3.9	6.9
Weight loss (kg)							
Pattern 1	9.4	6.2	5.5	7.7	11.1	1.2	52.3
Pattern 2	5.8 *	3	3.8	5	7.6	0.5	15.6
Pattern 3	7.1 *	4.7	3.8	6.2	9.3	−1.0	26.2
Pattern 4	2.3 *	1.5	0.9	2.6	3.4	0.4	4.6
Pattern 5	−0.2 *	1.2	−0.9	−0.1	0.3	−3.7	1.5
Pattern 6	0.9 *	1	0.3	0.9	1.3	−0.5	2.9
Pattern 7	−2.4 *	0.2	−2.5	−2.4	−2.3	−2.5	−2.3
Patterns 4–7	0.6 *	1.7	−0.3	0.5	1.4	−3.7	4.6
Initial weight (kg)							
Pattern 1	89.8	22.8	73.9	84.3	100.4	51.7	186
Pattern 2	91	21.3	77.6	88	101	55.6	163.1
Pattern 3	89.9	23	74.7	86.6	99.9	50.3	242.5
Pattern 4	80.6	19.7	63	73.9	96.4	60.3	114.8
Pattern 5	84.5	17.3	72.3	79.2	92.2	63.5	116.1
Pattern 6	71.7	18	54.9	73.6	85	48.1	98.4
Pattern 7	82.6	8.3	79.6	82.6	85.5	76.7	88.5
Patterns 4–7	80.5	18.1	67.3	77.3	91.2	48.1	116.1
Duration (days)							
Pattern 1	114.6	52.4	82	91	124.5	77	354
Pattern 2	116.4	55.7	81	90	131.5	77	336
Pattern 3	153.0 *	76.5	89.5	125	195	77	365
Pattern 4	105.6	47.7	80	86	108	77	244
Pattern 5	141.9	81.6	80.5	97	176.5	77	336
Pattern 6	83.6	6.3	80.5	82	82.8	77	95
Pattern 7	178	135.8	130	178	226	82	274
Patterns 4–7	120.1	68.3	80	85	131	77	336
Longest interval (days)							
Pattern 1	7.4	6.6	3	5.5	10	1	29
Pattern 2	9.2	6.7	4	8	13	1	28
Pattern 3	10.8 *	7.7	5	10	15	1	29
Pattern 4	9.2	7.8	2	10	13	1	29
Pattern 5	18.2 *	9.6	9.5	20	26.3	1	29
Pattern 6	8.9	8.6	3	6.5	12	1	29
Pattern 7	25.0 *	5.7	23	25	27	21	29
Patterns 4–7	13.8 *	9.9	5	13	25	1	29
Number of records							
Pattern 1	95	49.4	69.8	80	106.3	15	348
Pattern 2	88.3	46.9	62	78	100	20	290
Pattern 3	118.5 *	68.9	71	96	155	22	357
Pattern 4	79.2	30.7	54	76	100	39	132
Pattern 5	90.4	65.8	40	68	122.8	18	242
Pattern 6	60.1	17.1	46	66	73.3	32	81
Pattern 7	56.5	58.7	35.8	56.5	77.3	15	98
Patterns 4–7	78.9	49.3	46	68	98	15	242

* Indicates statistical significance: *p* < 0.05, reference group: Pattern 1.

**Table 6:** Results of the multinomial logistic regression.

Pattern	Term	OR	Lower CI	Upper CI
2	Weight change rate the first 14 days	0.7319	0.2630	2.0369
	Number of records in the first 14 days	0.9703	0.9000	1.0462
	Duration	1.0005	0.9959	1.0052
3	Weight change rate in the first 14 days	0.3775	0.0874	1.6305
	Number of records in the first 14 days	1.0183	0.9574	1.0830
	Duration	1.0094	1.0061	1.0126
4–7	Weight change rate in the first 14 days	0.0004	<0.0001	0.0159
	Number of records in the first 14 days	1.0567	0.9402	1.1876
	Duration	1.0001	0.9932	1.0071

## Data Availability

The data described in the manuscript, code book, and analytic code will be made available upon request, pending application and approval.
